# Novel α-Tubulin Mutations Conferring Resistance to Dinitroaniline Herbicides in *Lolium rigidum*

**DOI:** 10.3389/fpls.2018.00097

**Published:** 2018-02-06

**Authors:** Zhizhan Chu, Jinyi Chen, Alex Nyporko, Heping Han, Qin Yu, Stephen Powles

**Affiliations:** ^1^College of Life Sciences, South China Agricultural University, Guangzhou, China; ^2^Australian Herbicide Resistance Initiative, School of Agriculture and Environment, University of Western Australia, Perth, WA, Australia; ^3^Institute of High Technologies, Taras Shevchenko National University of Kyiv, Kiev, Ukraine

**Keywords:** dinitroanilines, trifluralin resistance, α-tubulin, mutation, *Lolium rigidum*

## Abstract

The dinitroaniline herbicides (particularly trifluralin) have been globally used in many crops for selective grass weed control. Consequently, trifluralin resistance has been documented in several important crop weed species and has recently reached a level of concern in Australian *Lolium rigidum* populations. Here, we report novel mutations in the *L. rigidum* α-tubulin gene which confer resistance to trifluralin and other dinitroaniline herbicides. Nucleotide mutations at the highly conserved codon Arg-243 resulted in amino acid substitutions of Met or Lys. Rice calli transformed with the mutant 243-Met or 243-Lys α-tubulin genes were 4- to 8-fold more resistant to trifluralin and other dinitroaniline herbicides (e.g., ethalfluralin and pendimethalin) compared to calli transformed with the wild type α-tubulin gene from *L. rigidum*. Comprehensive modeling of molecular docking predicts that Arg-243 is close to the trifluralin binding site on the α-tubulin surface and that replacement of Arg-243 by Met/Lys-243 results in a spatial shift of the trifluralin binding domain, reduction of trifluralin-tubulin contacts, and unfavorable interactions. The major effect of these substitutions is a significant rise of free interaction energy between α-tubulin and trifluralin, as well as between trifluralin and its whole molecular environment. These results demonstrate that the Arg-243 residue in α-tubulin is a determinant for trifluralin sensitivity, and the novel Arg-243-Met/Lys mutations may confer trifluralin resistance in *L. rigidum*.

## Introduction

Dinitroaniline herbicides have been widely used for selective grass weed control in many crops. Trifluralin is a soil-applied, pre-emergence dinitroaniline herbicide long used in Australia, where innovations in seeding machinery have enabled trifluralin to be safely used at a relatively high rate (>960 g ha^-1^) in no-till/conservation farming systems. In these systems, trifluralin controls germinating grass weed seeds in wheat and other crops. Nationally, trifluralin sustainability is important as trifluralin controls grass weeds that have evolved resistance to post-emergence herbicides (e.g., acetolactate synthase (ALS)- and acetyl coenzyme A carboxylase (ACCase)-inhibitors). Trifluralin resistance in *Lolium rigidum* (annual ryegrass), the most problematic and herbicide resistance-prone weed of Australian cropping systems, was first reported two decades ago (McAlister et al., 1995), and in recent years significant levels of trifluralin resistance in *L. rigidum* have been identified across large areas of Australian crop land (Owen et al., 2007, 2014; Boutsalis et al., 2012).

Alpha- and β-tubulin are cytosolic proteins which assemble into dynamic structures known as microtubules, essential for cell expansion and division (Howard and Hyman, 2003). Dinitroaniline herbicides (e.g., trifluralin, pendimethalin and ethalfluralin) bind to tubulin and interrupt the polymerisation of microtubules, arresting cell division and elongation, and resulting in plant death. Although the precise tubulin binding sites for the dinitroanilines have not been determined, molecular structural modeling, together with analysis of tubulin resistance mutations in plants and protozoa (see below), has demonstrated that dinitroanilines most likely interact with α-tubulin to disrupt microtubule polymerisation (Blume et al., 2003; Morrissette et al., 2004; Nyporko and Blume, 2008, 2014; Nyporko et al., 2009), although β-tubulin may also be a target (Blume et al., 1998; Nyporko and Blume, 2008).

Despite repeated use of dinitroaniline herbicides, especially trifluralin, worldwide, resistance to dinitroanilines is still relatively rare (12 reported resistance cases) as compared to other major herbicides (e.g., 158 cases of resistance to ALS-inhibiting herbicides, 47 to ACCase-inhibiting herbicides, and 34 to glyphosate) (Heap, 2017). Internationally, trifluralin resistance has been thoroughly investigated in only two (self-pollinated) weed species, *Eleusine indica* and *Setaria viridis*. Resistance was due to target-site α-tubulin mutations of Thr-239-Ile and Met-268-Thr in *E. indica* (Anthony et al., 1998; Yamamoto et al., 1998), and of Leu-136-Phe and Thr-239-Ile in *S. viridis* (Délye et al., 2004). In addition, Val-202-Phe and Leu-125-Met/Leu-136-Phe mutations were identified in resistant *Alopecurus aequalis* plants (Hashim et al., 2012). Therefore, a small number of specific tubulin gene mutations have been confirmed to endow resistance to dinitroaniline herbicides in weedy plants. In contrast, chemical mutagenesis studies in Protozoan parasites (e.g., *Toxoplasma gondii*) have identified many α-tubulin gene mutations (e.g., at positions His-8, Leu-136, Ser-165, Thr-239, Arg-243, Val-252 and Met-301) that confer high level resistance to dinitroanilines (Morrissette et al., 2004; Nyporko and Blume, 2008; Lyons-Abbott et al., 2010). In addition, early chemical mutagenesis studies in the green alga *Chlamydomonas reinhardtii* identified an α-tubulin gene mutation of Tyr-24-His and a β-tubulin gene mutation of Lys-350-Glu/Met which conferred resistance to dinitroanilines and colchicine/dinitroanilines, respectively (Lee and Huang, 1990; James et al., 1993). Fitness studies of the α-tubulin mutations revealed that a moderate fitness penalty was associated with some tubulin mutations (e.g., Thr-239-Ile, Darmency et al., 2011), and this may explain the relatively low frequency of dinitroaniline herbicide resistance in weeds. Similarly, it was found that various α-tubulin mutations (including above-mentioned mutations at positions 136, 239, 243, and 268) in the parasite *T. gondii* confer dinitroaniline resistance at a cost to microtubule function (Ma et al., 2007).

Understanding the molecular basis of trifluralin resistance in *L. rigidum* has been only a recent event with identification of the two α-tubulin mutations of Val-202-Phe and Thr-239-Ile (Chen et al., 2017; Fleet et al., 2017). The current research employed molecular, transgenic and structural modeling approaches to reveal novel α-tubulin gene mutations endowing dinitroaniline herbicide resistance in *L. rigidum.*

## Materials and Methods

### Plant Material and Herbicide Treatment

A trifluralin-resistant *L. rigidum* population (M4/16; hereafter referred to as R) was originally collected from the Western Australian grain belt in 2010 (Owen et al., 2014). This population also displays cross-resistance to other dinitroaniline herbicides (Chen et al., 2017). A well-characterized herbicide-susceptible population (VLR1; hereafter referred to as S) was used as a control. Seeds of the R and S populations were germinated on moist filter paper at room temperature for 2–3 days, and germinating seeds (with the radicle just visible) transplanted 1 cm deep in plastic pots containing commercial potting mix (50% peatmoss, 25% sand and 25% pine bark). Trifluralin rates of 960–1920 g ha^-1^ achieves 100% control of the S population (Chen et al., 2017), and these rates were applied with a cabinet sprayer delivering the herbicide in 118 L ha^-1^ water through a dual flat-fan nozzle (TeeJet XR11001) in two passes at 200 kPa. Immediately after treatment, a 1 cm layer of moist potting mix was placed over the seeds. The pots were kept in a naturally lit glass house during the normal winter growing season (May–September). Seedling emergence and survivorship was determined 3 weeks after treatment, and plants that emerged from the soil and produced healthy tillers were considered to be trifluralin-resistant survivors.

### Alpha-Tubulin Gene Sequencing and Cloning

Total RNA was isolated from R individuals that survived the trifluralin field rate (960–1920 g ha^-1^), and from untreated S plants, using the ISOLATE II RNA Plant Kit (Bioline). Genomic DNA was removed using the TURBO-DNA free kit (Ambion) according to the manufacturer’s instructions. Total RNA (2 μg) were used for cDNA synthesis with the SuperScript^®^III reverse transcriptase (Invitrogen). The primer pair of ATG (5′-ACCATGAGGGAGTGCATCTCG-3′) and UAG (5′-GCTCTAGTACTCATCACCCTC-3′) (annealing at 56°C), together with an internal sequencing primer 5-R-sequence (5′-CAGGCCATGTACTTGCCGTG-3′) were used to amplify and sequence a 1356 bp α-tubulin fragment containing known resistance mutation sites. An additional primer pair of A4F2 (5′-ACCGCAGGGACAGGCGTCTTCGTAC-3′) and A4R2 (5′-CTGTGCTATGTTTGAACCTGCTTCG-3′) (annealing at 56°C) were designed in the 5′- and 3′-UTRs for cloning the full coding sequences of the amplified α-tubulin alleles into the pGEM-T vector (Promega). All primers were designed based on published *L. rigidum* gene sequences (Duhoux et al., 2015)^1^ (accessions SRR1141056 to SRR1141083) as well as tubulin sequences from our own unpublished *L. rigidum* transcriptome, and α-tubulin sequences of other weed species (e.g., *E. indica, S. viridis, A. aequalis*). The PCR conditions were: 95°C for 3 min, then 31 cycles of 95°C for 30 s, X°C (annealing temperature) for 30 s and 72°C for 1 min, followed by a final extension step of 7 min at 72°C.

### Rice Callus Transformation and Growth Response to Trifluralin

**FIGURE 1:**
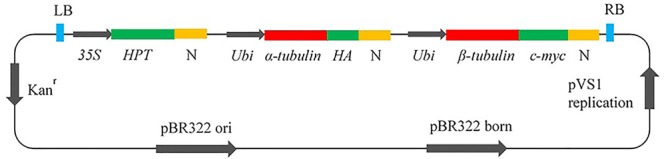
Vector construct for co-expression of α- and β-tubulin genes in rice callus. *HPT*, hygromycin phosphotransferase; N, Nopaline synthase terminator; *Ubi*, Ubiquitin promotor; *HA*, Hemagglutinin epitope tag; c-myc, epitope tag; Kan, kanamycin.

To achieve cell viability, it is essential to co-express both α- and β-tubulin genes (Anthony and Hussey, 1998); therefore, DNA cassettes were constructed as in **Figure 1**. The inserts contained the hygromycin phosphotransferase (HPT) gene for transformation selection under the control of the 35S promoter; the wild–type (WT) (Arg-243), or mutant (Met-243, Lys-243) α-tubulin cDNA from *L. rigidum* and the wild type β-tubulin coding sequence (Os05g0413200) from *Oryza sativa* (Japonica Nipponbare); the ubiquitin (Ubi) promoter; and the nopaline synthase (N) terminator. Hemagglutinin (HA) and c-myc (a transcription factor) epitope tags for antibody binding were fused to α- and β-tubulin respectively. The vectors were introduced into *Agrobacterium tumefaciens* by electroporation and the transformed *A. tumefaciens* strains were used to transform WT Nipponbare rice. All constructed vectors were checked carefully by restriction analysis and DNA sequencing before being used for rice transformation.

Hygromycin-resistant rice calli were selected and sub-cultured on the NB-medium (3% sucrose, 0.03% casamino acids, 0.4% N6 salts, 0.29% proline, 0.2% 500× N6 vitamin, 0.03% 10 g L^-1^ 2,4-D and 0.4% gelrite, pH = 5.8) plates containing 0.05 g L^-1^ hygromycin and 0.3 g L^-1^ carbenicillin, and proliferating calli transferred onto fresh NB plates containing trifluralin, ethalfluralin or pendimethalin at 0, 0.5, 2, 4, 6, 8, and 10 mg L^-1^ (stock solution prepared in DMSO, and the control solution contained the same amount of DMSO). Herbicide concentrations at ≥0.5 mg L^-1^ inhibited growth of the non-transgenic rice calli. For each herbicide concentration, 10 transformed calli were used, and at least three independent transformation experiments were conducted. After 2 weeks in the dark, growth response to the three herbicides was compared between calli transformed with the WT and mutant recombinant vectors. Other hygromycin-resistant and herbicide-untreated calli were transferred to differentiation and rooting medium to obtain transformed WT and mutant rice seedlings for the immunoblot analysis. The introduction of the transgene into rice calli and seedlings was confirmed by PCR using the forward primer TUBUF (5′-TATGGAGGAGGGAGAGTT-3′) and the reverse primer TUBUR (5′-TCTGGAACATCGTATGGG-3′) which spans both the α-tubulin and the vector sequences of the HA tag.

### Immunoblot Analysis

Immunoblot detection of the HA tag by enhanced chemiluminescence (ECL) was performed to assess the production of transgenic α-tubulin according to Zhao et al. (2015). In brief, tissue of rice leaves (300 mg) was ground into powder in liquid nitrogen and lysed in 1 mL of TBST buffer (0.1% Tween-20, 100 mM Tris-HCl, and 150 mM NaCl, pH 7.5). After centrifugation at 13,400 *g* for 10 min, the supernatant was mixed with an equal volume of 2× loading buffer (100 mM Tris-HCl, 5 mM DTT, 4% SDS, 0.01% bromophenol blue, and 30% glycerol, pH 6.8) and heated for 3 min at 90°C. The total proteins (75 μg) were separated on 12% SDS-PAGE gels, and then transferred onto a PVDF membrane using a Bio-Rad mini transfer cell. The membrane was blocked in TBST buffer containing 5% (w/v) milk powder and 0.1% Triton X-100, washed with fresh TBST buffer, and probed with a 1:10,000 dilution of mouse anti-heat-shock protein 70 (HSP) or rabbit anti-HA primary antibody (TransGen Biotech, China). After rinses with TBST, the membranes were incubated in 1:10,000-diluted secondary antibody solution (horseradish peroxidase-conjugated goat anti-mouse or goat anti-rabbit IgG (H + L) for 2 h at room temperature, and washed twice with TBST. The membrane blots were incubated in the ECL substrate for 2 min, and the immunoblotting signals detected by chemiluminescence (Tanon 5200, Bio-Tanon, China). Protein extracts from the non-transgenic rice seedlings were used as a negative control, and the signals from HSP as a loading control.

### Structural Modeling of α-Tubulin Variants

The spatial structure of the WT isoform of *L. rigidum* α-tubulin was reconstructed by a homology modeling approach (Venselaar et al., 2010) using the MODELLER software (Eswar et al., 2006). The previously obtained spatial structures of α-tubulins from *Arabidopsis thaliana* (Blume et al., 2010; Nyporko and Blume, 2014) and *E. indica* (Nyporko et al., 2002, 2009) were used as templates for *L. rigidum* α-tubulin reconstruction. To generate the promodels of mutant *L. rigidum* α-tubulin isoforms, the appropriate amino acids at position 243 (Arg-Met, Arg-Lys) were changed using the Discovery Studio Visualizer software version 4.5.^2^ Spatial geometry of obtained promodels of the WT and mutant α-tubulin isoforms was optimized via energy minimization using the L-BFGS algorithm (Das et al., 2003) and combined CHARMM force field (CHARMM27 with implemented CMAP) (MacKerell et al., 2000, 2004) with the gmx *mdrun* module of GROMACS software (Abraham et al., 2015). The maximum number of optimization steps was 1000. Convergence criteria were adopted by setting the maximum force on the atoms emtol to 10 kJ mol^-1^ nm^-1^ and using a maximum step size of 0.01 nm. Structural differences between the WT and mutant isoforms were analyzed using the Swiss-PDB Viewer software.

Docking the trifluralin molecule into the α-tubulin surface was performed with the S4MPLE software (Hoffer and Horvath, 2012). The topology of trifluralin for application in molecular dynamics (MD) simulations was performed via the web-based tool Swiss Param (Zoete et al., 2011). To simulate realistic intracellular conditions, all further calculations were carried out in a water solution containing 0.15 M NaCl. The prepared complexes were placed in a rectangular periodic box using the gmx *editconf* module of the GROMACS software. Thereafter, the box was filled by water molecules (using the gmx *solvate* module), and Na^+^ and Cl^-^ ions were added into the system (using the gmx *genion* module). The properties of the water molecules were calculated using the standard TIP3 model (Mahoney and Jorgensen, 2000). The obtained systems were subjected to energy minimization procedures, as described above.

In the optimized systems, we calculated the position restrained MD within a 100 ps interval (to achieve the equilibrate state) and the unrestrained (productive) MD within a 100 ns time interval at 300K. The initial velocities were generated according to the Maxwell–Boltzmann distribution at 300K. A classical leap-frog algorithm was applied for the integration of the motion equations (Hockney et al., 1974), electrostatic interactions calculated using the particle mesh Ewald method (Darden et al., 1993), and Van der Waals interactions accounted for by using the cut-off radius method. The temperature and pressure of the system were controlled using a V-rescale thermostat (Bussi et al., 2007) and a Parinello-Rahman barostat, respectively. No error messages or unacceptable steric parameters occurred in the systems during the calculation procedures. All the energy parameters of the investigated system, and its components, were calculated using the *gmx energy* module. Free interaction energy was calculated using the g_mmpbsa tool (Kumari et al., 2014).

## Results

### Alpha-Tubulin Gene Sequencing Identified Arg-243-Met/Lys Amino Acid Substitutions

To identify the molecular basis for target-site based trifluralin resistance in the resistant *L. rigidum* population, a 1356 bp full-length α-tubulin coding sequence was amplified and analyzed from trifluralin-resistant (R) individuals (surviving 960 and 1920 g ha^-1^ trifluralin) and compared to individuals from the susceptible (S) population. Of the R plants analyzed, 15% had nucleotide mutations at codon 243 (AGG to AAG, or AGG to ATG), resulting in amino acid substitutions of Arg-243-Met or Arg-243-Lys. However, most of the Arg-243 mutations occurred in combination with previously identified Thr-239-Ile (Anthony et al., 1998) or Val-202-Phe (Hashim et al., 2012) mutations. The percentage of plants homozygous for the Arg-243-Lys mutation was low, only accounting for 2.6% of the 39 surviving plants analyzed, and no plants homozygous for the Arg-243-Met mutation were found. An additional 22 untreated plants from the R population were analyzed and again no homozygous Arg-243-Met or Arg-243-Lys mutation was detected. The 1356 bp full cDNA coding sequences from WT, 243-Met and 243-Lys mutants, encoding an identical α-tubulin isoform (451-amino acids), only differing at the 243 codon, were cloned and used for subsequent rice genetic transformation.

### Rice Calli Transformed with *L. rigidum* 243 Mutant Alleles Exhibit Resistance to Trifluralin and Other Dinitroaniline Herbicides

**FIGURE 2:**
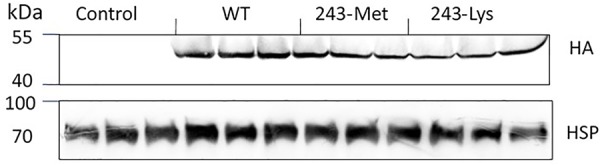
Western blot analysis of expression of WT, and mutant 243-Met and 243-Lys α-tubulin variants in transgenic rice seedlings in comparison to an untransformed control, probed by antibody against fused HA (hemagglutinin) epitope tag. HSP (Heat shock protein 70) expression was used as a protein loading control.

**FIGURE 3:**
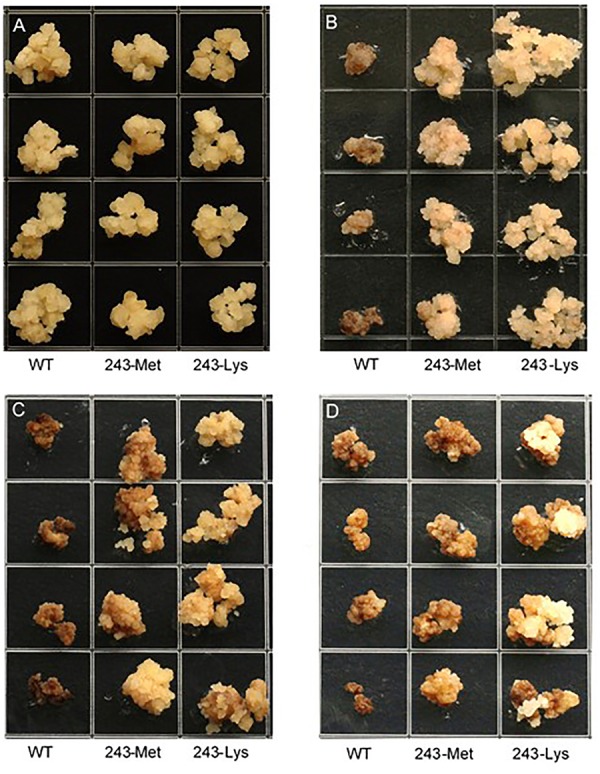
Growth of rice calli transformed with the WT, mutant 243-Met or 243-Lys α-tubulin genes in medium containing **(A)** 0, **(B)** 0.5, **(C)** 2.0, and **(D)** 4.0 mg L^-1^ trifluralin.

**FIGURE 4:**
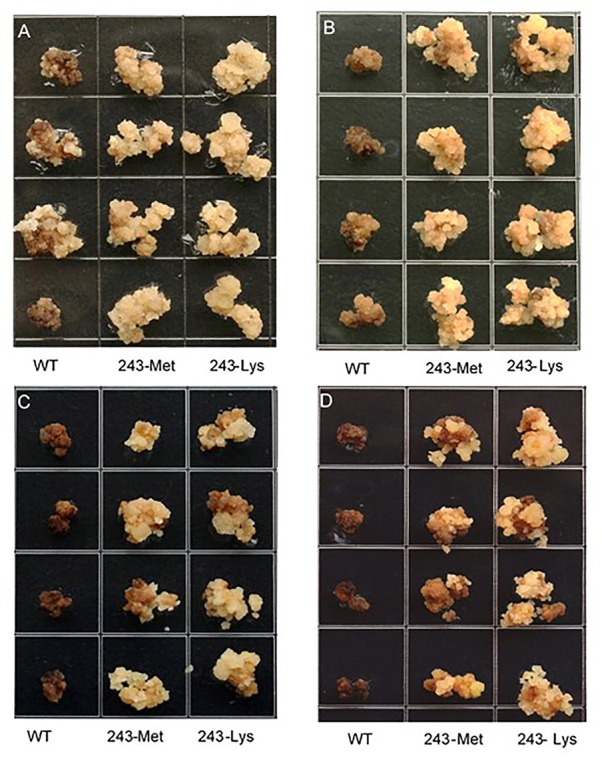
Growth of rice calli transformed with the WT, mutant 243-Met or 243-Lys α-tubulin genes in medium containing **(A)** 2.0, **(B)** 4.0, **(C)** 6.0, and **(D)** 8.0 mg L^-1^ ethalfluralin.

**FIGURE 5:**
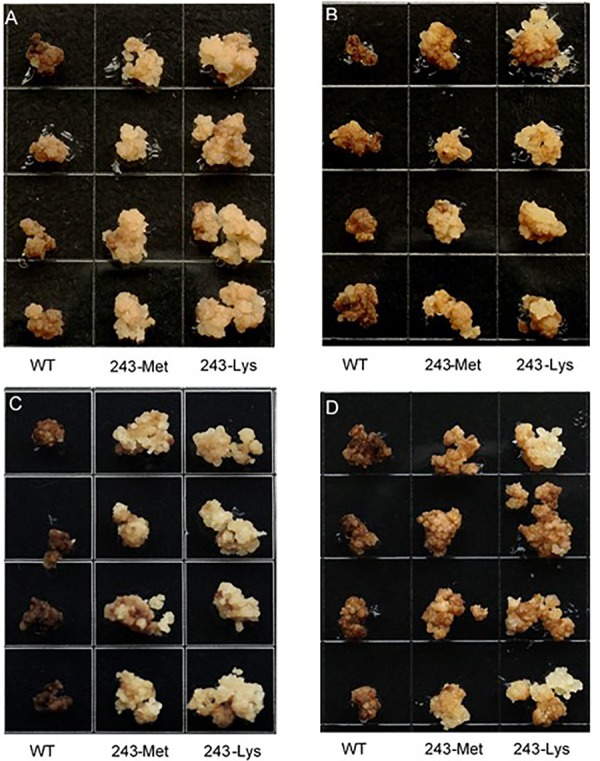
Growth of rice calli transformed with the WT, mutant 243-Met or 243-Lys α-tubulin genes in medium containing **(A)** 2.0, **(B)** 4.0, **(C)** 6.0, and **(D)** 10 mg L^-1^ pendimethalin.

Three *L. rigidum* α-tubulin variants (WT, 243-Met and 243-Lys) were successfully introduced into rice calli, and their expression in transgenic rice calli and the T_0_ seedlings was confirmed by PCR (not shown) using the primer pair TUBUF/TUBUR, and by immunoblot analysis, respectively (**Figure 2**). Growth of the untransformed rice calli was used to determine the minimum concentration of each herbicide needed to inhibit rice callus growth (0.5 mg L^-1^ for trifluralin, 2.0 mg L^-1^ for ethalfluralin and pendimethalin, respectively). Proliferation of the rice calli transformed with WT, 243-Met and 243-Lys gene variants were compared on growth medium containing increasing concentrations of trifluralin or other dinitroaniline herbicides. The transformed WT calli were susceptible to trifluralin, and did not grow at trifluralin concentrations ≥ 0.5 mg L^-1^ (**Figure 3B**). However, a trifluralin concentration of 4 mg L^-1^ (i.e., 8-fold higher) was required to stop proliferation of the transformed 243-Met and 243-Lys calli (**Figures 3B–D**). Similarly, 4- to 5-fold higher concentrations of the dinitroaniline herbicides ethalfluralin and pendimethalin were required to inhibit the growth of transformed 243-Met or 243-Lys rice calli, relative to the transformed WT calli (**Figures 4, 5**). This clearly demonstrates that the Arg-243-Met/Lys mutations endow resistance to dinitroaniline herbicides, with the Arg-243-Lys mutant being more resistant than the Arg-243-Met mutant (**Figures 3–5**).

### 3D Molecular Modeling Reveals Structural Interactions of the *Lolium* Mutant α-Tubulin and Trifluralin

**FIGURE 6:**
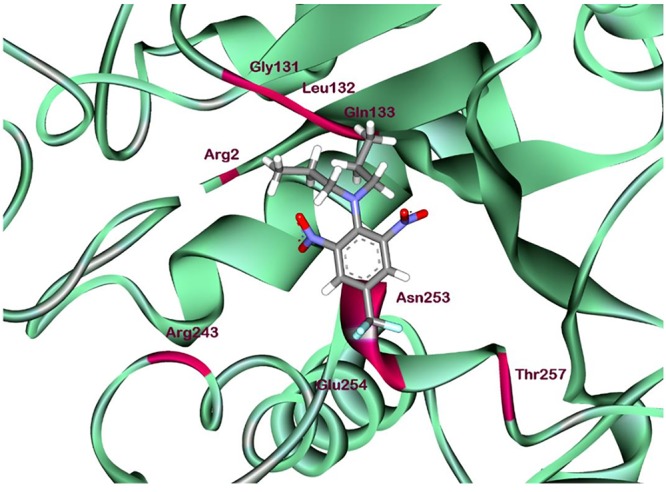
Ribbon diagram view of predicted trifluralin-binding amino acids relative to the position of amino acid Arg-243 in *Lolium rigidum* α-tubulin. Positions of binding site amino acids and Arg-243 (arrowed) are colored by burgundy. Trifluralin atoms colored by standard color scheme for atom representation.

**FIGURE 7:**
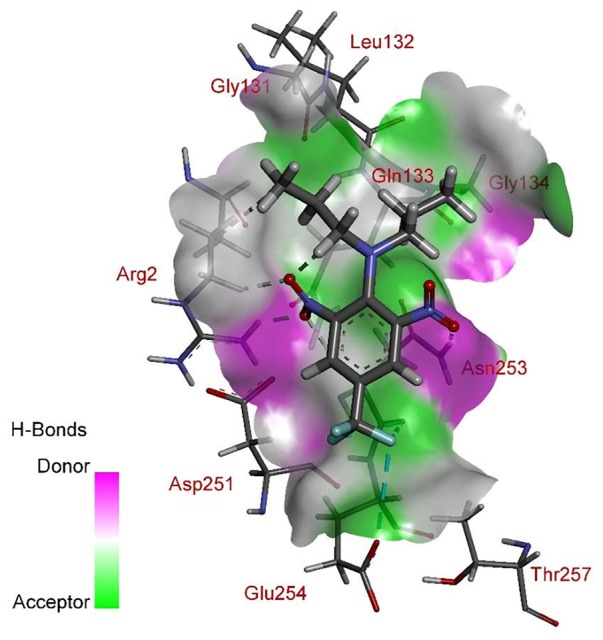
Spatial structure of contact interface between trifluralin and wild type (WT) α-tubulin. Protein contact surface is colored by H-bond donor/acceptor distribution, binding site amino acids represented by sticks, and intermolecular contacts indicated by dotted lines.

According to comprehensive molecular docking simulations, trifluralin is able to bind to *L. rigidum* α-tubulin in two different models. In the first binding model, the trifluralin binding site on the α-tubulin surface consists of amino acid residues Arg-2, Gly-131, Leu-132, Gln-133, Asn-253, Glu-254, and Thr-257 (**Figures 6, 7**). During the studied MD interval, the residues Gly-134 and Asp-251 may also have temporary interactions with the trifluralin molecule (**Figure 7**). In the starting geometry the ligand (trifluralin) forms two conventional hydrogen bonds in the amino acid environment, as well as two carbon-hydrogen bonds. One conventional hydrogen bond is located between the hydrogen atom of a side chain imide (guanidine) group of Arg-2, and the oxygen atom of the first trifluralin nitro group; and the other is formed between the hydrogen atom of the Asn-253 amide group and the oxygen atom of the second trifluralin nitro group (**Figure 7**). The same amino acids (Arg-2 and Asn-253) are also involved in the formation of two carbon-hydrogen bonds. One carbon-hydrogen bond is located between the hydrogen atom of the Arg-2 side chain, and the oxygen atom of the first trifluralin nitro group, and the other is between the hydrogen atom of the trifluralin hydrocarbon radical and the oxygen atoms of the Asn-253 side chain amide group.

As a result of 100 ns MD modeling, the trifluralin molecule reorientates on the surface of the α-tubulin binding site. In the final conformation (state), trifluralin forms five hydrogen bonds (four conventional hydrogen bonds and one carbon-hydrogen bond). Three of the conventional hydrogen bonds are located between the fluorine atoms of the trifluralin trifluoromethyl group and the hydrogen atoms of the nitro groups of the Gln-133, Asn-253 and Glu-254 residues. The fourth conventional hydrogen bond is located between the hydrogen of the Tyr-257 hydroxyl group and the oxygen of the trifluralin nitro group. The carbon-hydrogen bond involves the hydrogen of the trifluralin hydrocarbon radical and the oxygen of the Asn-253 side chain amide group, which coincides with the same hydrogen bond formed at the starting conformation of the trifluralin-tubulin complex.

A second possible binding model is composed of residues Met-1, Arg-2, Gly-34, Leu-242, Arg-243, Phe-244, Asp-245, Gly-246, Asn-249 and Asp-251 (not shown). Trifluralin forms five conventional and four carbon-hydrogen bonds with α-tubulin in the starting geometry of the complex. However, during 100 ns MD, the trifluralin molecule practically detaches from the binding area and remains in contact with only two amino acid residues. Correspondingly, the average potential energy of trifluralin-α-tubulin interaction over the 100 ns time interval is low (-159.26 kJ mol^-1^) for the first model, and high (-61.03 kJ mol^-1^) for the second model. Thus, the second model is considered to be unrealistic and unstable, and was discarded.

**FIGURE 8:**
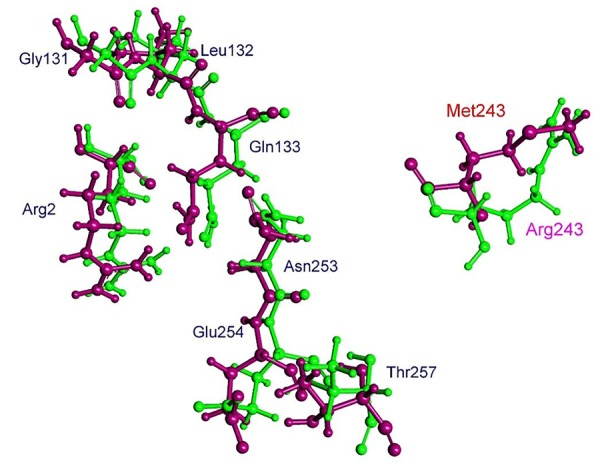
Spatial arrangement of trifluralin-binding amino acids in WT and the Arg-243-Met mutant α-tubulin isoform. Residues of WT and mutant isoforms are colored by green and violet, respectively.

**FIGURE 9:**
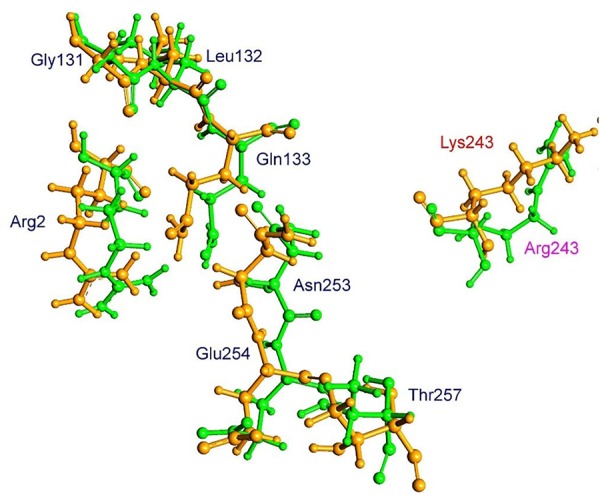
Spatial arrangement of trifluralin-binding amino acids in WT and the Arg-243-Lys mutant α-tubulin isoforms. Residues of WT and mutant isoforms are colored by green and orange, respectively.

**FIGURE 10:**
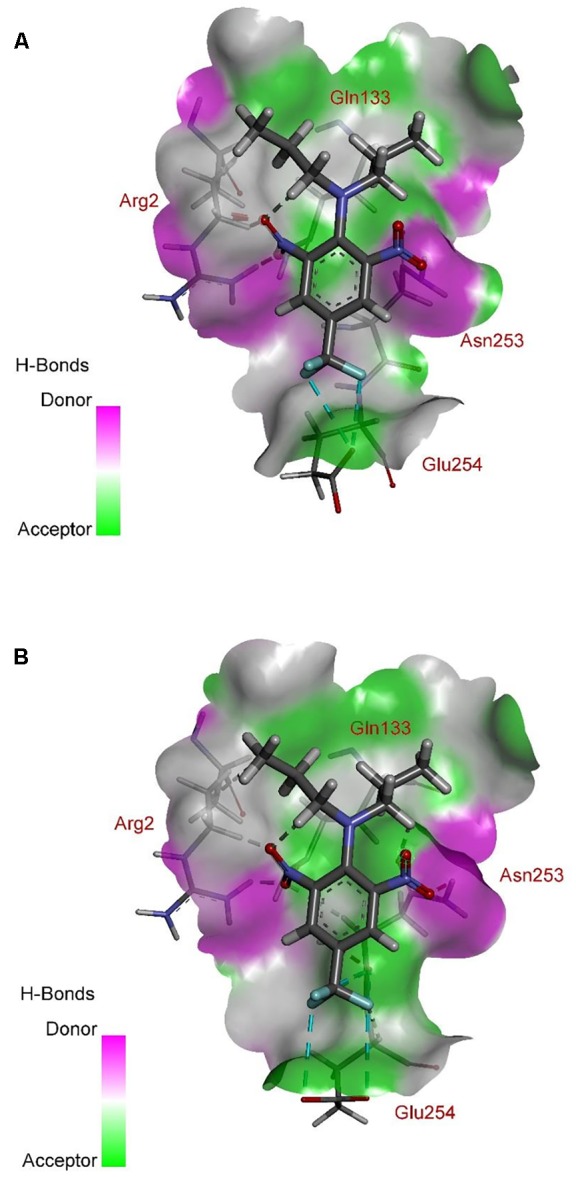
Spatial structure of contact interface between trifluralin and the Arg-243-Met **(A)**, and between trifluralin and the Arg-243-Lys **(B)** mutant α-tubulins. Protein contact surface is colored by H-bond donor/acceptor distribution, binding site amino acids represented by sticks, and intermolecular contacts indicated by dotted lines.

**FIGURE 11:**
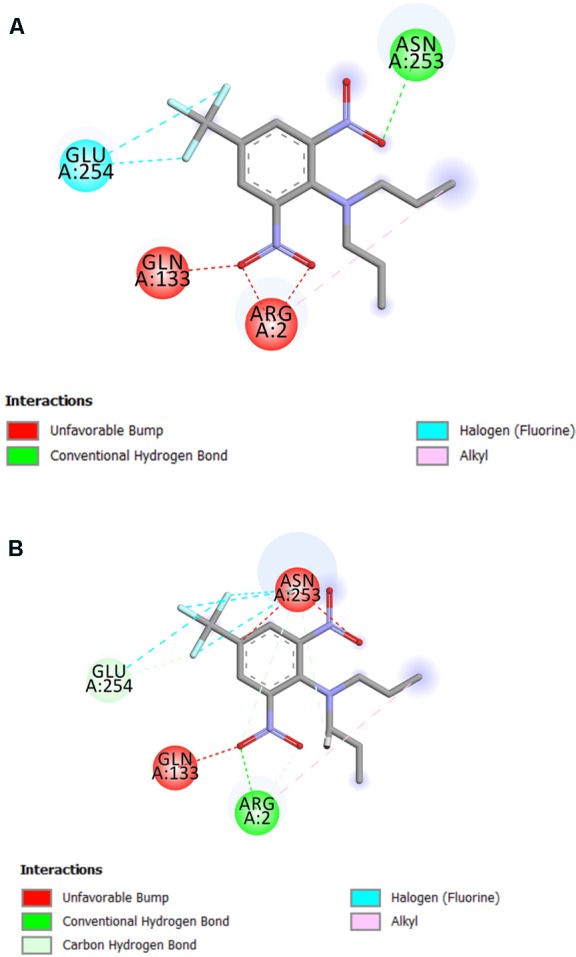
2D diagram of intermolecular interactions (shown by dotted lines) between trifluralin and the Arg-243-Met **(A)**, and between trifluralin and the Arg-243-Lys **(B)** mutant α-tubulins.

The residue Arg-243 directly contacts residues Arg-2 and Gln-133 in the trifluralin binding site, so that mutations in this position have an impact on its spatial structure, behavior and other functional properties. Both the Arg-243-Met and Arg-243-Lys mutations discovered in the current study resulted in a definite space shift of the protein main chain in the trifluralin binding area, and a reorientation of the appropriate side chains (**Figures 8, 9**), with significant root mean square deviation (RMSD) of 0.95 and 1.01 Å, respectively. The two 243 mutations also reduced the number of contact residues from nine in the WT tubulin (**Figure 7**) to only four in the mutant tubulin (**Figures 10A,B**). In addition, the two mutations caused unfavorable interactions between trifluralin and the tubulin. For example, the Arg-243-Met replacement clashes with residues Arg-2 and Gln-133 (**Figure 11A**), and the Arg-243-Lys substitution clashes with residues Gln-133 and Asn-253 (**Figure 11B**). The most important outcome of these substitutions was the 2.5- and 3.7-fold rise of free interaction energy between trifluralin and tubulin for 243-Met and 243-Lys isoform, respectively, as compared to that for the WT Arg-243 isoform (**Table 1**). In addition, the free interaction energy between trifluralin and the remainder of the molecular environment was also increased up to 1.6- and 1.75-fold for the 243-Met and 243-Lys isoforms, respectively, relative to that for the WT (**Table 1**). These results predict potential instability of both the 243-Met and the 243-Lys mutant tubulin complexes as compared to the WT. Thus, both of the amino acid substitutions detected at position 243 of *L. rigidum* α-tubulin are predicted to confer trifluralin resistance. Moreover, consistent with the results from transgenic rice calli, the above structural modeling analysis parameters also indicated a higher level of resistance to trifluralin endowed by the Arg-243-Lys mutation than by the Arg-243-Met mutation (**Table 1**).

**Table 1:** Estimation of free interaction energies for the wild type (WT) and mutant (Arg-243-Met and Arg-243-Lys) α-tubulin isoforms in *Lolium rigidum.*

Tubulin isoform	Free interaction energy between tubulin and trifluralin (kJ/mol)	Free interaction energy between trifluralin and the rest of molecular environment (kJ/mol)
WT (Arg-243)	–242	–421
Arg-243-Met	–96	–267
Arg-243-Lys	–66	–240

## Discussion

Trifluralin as a soil-applied, pre-emergence dinitroaniline herbicide was first introduced into Australia in the 1960s and extensively adopted. Trifluralin resistance was initially documented in *L. rigidum* in the 1990s (McAlister et al., 1995), but the molecular basis of resistance had remained unknown. In this research, we report two novel amino acid substitutions (Arg-243-Met and Arg-243-Lys) in the α-tubulin of a trifluralin-resistant *L. rigidum* population. Either of these two mutations confers resistance to dinitroaniline herbicides. The significance of the Arg-243 residue in dinitroaniline resistance is illustrated by the following: firstly, Arg-243 is highly conserved in all but one (the fungus *Histoplasma capsulatum*) of the more than 150 fungal, animal, protozoan and plant α-tubulin sequences deposited in SwissProt, indicating its importance in tubulin function (Nyporko and Blume, 2008). Secondly, Arg-243 has been predicted to be involved in trifluralin binding in the α-tubulin of the plant *E. indica* (Blume et al., 2003; Nyporko et al., 2009) and the protozoa *Toxoplasma gondii* and *Plasmodium falciparum* (Morrissette et al., 2004; Nyporko et al., 2009). In addition, as dinitroaniline herbicides are only active against plant and protozoa, but not against animal or fungal microtubules, tubulin mutations identified only in plants and protozoa are of high relevance. For example, chemical mutagenesis studies in the protozoan parasite *T. gondii* have identified various α-tubulin mutations (e.g., at positions His-8, Leu-136, Ser-165, Thr-239, Arg-243, Val-252, and Met-301) that confer high level resistance to the dinitroaniline herbicide oryzalin (Morrissette et al., 2004; Nyporko and Blume, 2008; Lyons-Abbott et al., 2010). Two α-tubulin mutations (Leu-136-Phe and Thr-239-Ile) that were identified in resistant *T. gondii* have also been identified in plants (Anthony et al., 1998; Yamamoto et al., 1998; Délye et al., 2004). In particular, amino acid substitutions introduced at Arg-243 (i.e., Arg-243-Cys/Ser) by chemical mutagenesis in protozoa resulted in α-tubulins that were highly resistant to oryzalin (Morrissette et al., 2004). The different mutations at the Arg-243 in *T. gondii* as compared to those in *L. rigidum* (in this current study) are related to a codon difference: the former is from CGT (Arg) to AGT (Ser)/TGT (Cys), and the latter is from AGG (Arg) to ATG (Met)/AAG (Lys). Based on the above information, the two amino acid substitutions identified in *L. rigidum* in the current study are highly likely to endow resistance to trifluralin and other dinitroaniline herbicides.

We took a transgenic approach to confirm if these tubulin mutations confer resistance when expressed in transgenic rice calli. Early research has established that co-expression of both α- and β-tubulins is a requirement for cell viability, as the plant cells cannot tolerate an imbalance in α- and β-tubulins (Anthony and Hussey, 1998). The first well-known α-tubulin mutation of Thr-239-Ile in plants that confers resistance to dinitroaniline herbicides was confirmed in transgenic maize calli (Anthony et al., 1998; Anthony and Hussey, 1999). Regenerable transgenic tobacco and finger millet (*Eleusine coracana*) seedlings overexpressing recombinant resistant α- and wild-type β-tubulins that are resistant to dinitroanilines have been obtained (Anthony et al., 1999; Bayer et al., 2014), demonstrating the relationship between the calli transformation and plant expression of the trangene. In the current study, we have successfully transformed rice calli with a mutant *L. rigidum* α-tubulin gene together with a wild-type rice β-tubulin gene. These rice calli transformed with the 243-Met or 243-Lys mutant α-tubulin allele displayed resistance to trifluralin and cross-resistance to other dinitroaniline herbicides, when compared to rice calli transformed with the WT allele (**Figures 3–5**). Production of the recombinant α- and β-tubulins in T_0_ rice seedlings was confirmed by the Western blot analysis (**Figure 2**). However, the T_1_ seeds of the 243 mutants, but not the transformed WT plants, are very poor in germination, and thus insufficient for further experiments. Among other reasons, this may be due to fitness penalty associated with the Arg-243-Lys/Met mutations (see below discussion on fitness cost).

Furthermore, using structural modeling, we illustrated how the Arg-243-Met and Arg-243-Lys mutations endow trifluralin resistance. Our modeling predicts that the Arg-243 residue is not directly involved in trifluralin binding, but is located close to the binding site (**Figure 6**). Replacement of Arg-243 by 243-Met or 243-Lys results in a spatial shift of the herbicide-binding domain (**Figures 8, 9**) and unfavorable interactions (**Figures 10, 11**). Predicted trifluralin binding sites on the surface of plant and protozoa α-tubulins are distinct but partially overlapping, sharing some residues (**Table 2**). Thus, all these sites can be considered as common binding sites, depending on the individual tubulin and/or bound herbicide molecule. Compared to the previous models of *E. indica* (Nyporko et al., 2002; Blume et al., 2003) and Arabidopsis (Blume et al., 2010; Nyporko and Blume, 2014), our current work shows that despite sharing a binding area, trifluralin can have different orientations in the binding site on the surface of different plant tubulins, and, correspondingly, various binding patterns involving different amino acid residues in the specific binding domain (Nyporko et al., 2009). Cross-resistance to the other dinitroaniline herbicides (ethalfluralin and pendimethalin) observed in this study is a persuasive proof of, firstly, the shared binding site by different dinitroanilines and, secondly, a “canonical” mechanism of dinitroaniline resistance, conferred by a single amino acid replacement (Nyporko and Blume, 2008; Nyporko et al., 2009). In addition, the most important impact of the observed amino acid substitutions in *L. rigidum*α-tubulin is the significant rise in the free interaction energy between α-tubulin and trifluralin, rendering trifluralin binding to α-tubulin unstable.

**Table 2:** Amino acid residues involved in trifluralin binding sites in plants (*Lolium rigidum, Eleusine indica*) and protozoa (*Plasmodium falciparum*) α-tubulins.

*L. rigidum*	*E. indica^∗^*	*P*. *falciparum^∗^*
**Arg-2, Gly-131,** Leu-132, **Gln-133**, Gly-134, **Asp-251, Asn-253, Glu-254, Thr-257**	**Arg-2, Gly-133, Arg-243, Asn-249,** Val-250, **Asp-251, Val-252, Asn-253, Gln-254**	**Arg-2, Gly-133, Arg-243, Asn-249,** Val-250, **Asp-251,** Val-252, **Thr-253, Gln-254**

*Contact residues are in bold font. ^∗^Nyporko et al. (2009)*.

Our 100 ns MD modeling also revealed that amino acid residues in the “second contact layer” (see Results), which includes Arg-243, are in direct contact with the trifluralin binding site and are all potential resistance-endowing mutation sites. In principle, it may be possible to predict all feasible sites of potentially resistance-endowing mutations, using this analysis of correlated motions in protein molecules, although this approach is not yet sufficiently robust.

In fact, all known mutations conferring resistance to antimicrotubular compounds are located close to specific binding sites (Nyporko and Blume, 2008). Several amino acid residues in the nearest environment of the predicted trifluralin binding site have been previously shown to be responsible for changes of plant cell sensitivity/resistance to dinitroaniline compounds (see Nyporko et al., 2009 for review). Classic amino acid replacements in positions 136, 239, and 268 that endow dinitroaniline resistance in weedy species (Anthony et al., 1998; Yamamoto et al., 1998; Délye et al., 2004) are located sufficiently close to the trifluralin binding site and/or the “second contact layer” of the *L. rigidum* α-tubulin model. As an additional proof, the Arg-243 was also found to be a part of the “second layer” in *L. rigidum* α-tubulin-trifluralin binding model.

Replacement of amino acids in the contact interface will not obligatorily disrupt the interaction between the tubulin subunits and, consequently, these replacements may avoid a major resistance cost. However, the Arg-243 residue is involved in forming a longitudinal contact interface, by directly interacting with Glu-71 of the β-subunit during certain stages of the microtubule life cycle, based on the data deposed in Protein Data Bank by (Alushin et al., 2014). Also, the normal Arg-243 residue in α-tubulin densely contacts with residues Thr-51 and Phe-52, which are possibly involved in the lateral contact interface (Nogales, 1999). Therefore, it is reasonable to assume that mutations in position 243 can influence these residues’ conformation and, thus, alter the spatial structure of this interface. It, in turn, could result in abnormal tubulin polymerization, especially when the ratio of mutant versus normal (WT) tubulin is high (e.g., in homozygous mutants as discussed below).

**FIGURE 12:**
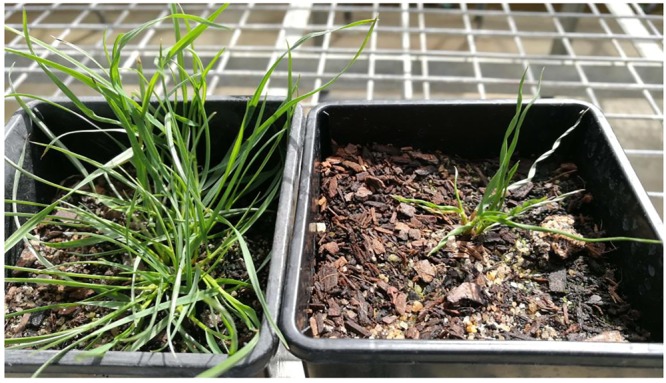
Helical growth of the 243-Met mutant (right side) as compared to the WT (left side).

In fact, we noticed that in the resistant *L. rigidum* population, the percentage of plants homozygous for the Arg-243-Lys mutation was low (2.6%), and we did not find plants homozygous for the Arg-243-Met mutation (61 plants analyzed). The possible reasons are (1) these two mutations are recent mutational events, and (2) these two mutations endow a fitness cost when in the homozygous state, by affecting normal tubulin function. A moderate fitness cost (as 20% yield loss) has been reported for the homozygous Thr-239-Ile mutation in *Setaria* (Darmency et al., 2011). Similarly, various α-tubulin mutations (including the above-mentioned mutations at positions 136, 239, 243, and 268) in the parasite *T. gondii* confer dinitroaniline resistance at a cost to microtubule function (Ma et al., 2007). In conditions when we forced heterozygote Arg-243-Met mutant plants to cross with each other, the resultant homozygous mutant plants showed right-hand helical twisting (**Figure 12**). Compared with the normal WT plant, the homozygous Arg-243-Met mutant grew very slowly (**Figure 12**). This is very interesting not only in understanding the potential fitness cost of resistance, but also the mechanisms controlling dwarfism. Similar effects have been found in rice and Arabidopsis mutants homozygous for the Thr-56-Ile or Ser-180-Phe mutation in the α-tubulin gene (Sunohara et al., 2009). Further research into these homozygous *L. rigidum* plants is needed to confirm the causal link between the Arg-243-Met mutation and the observed helical phenotype.

## Conclusion

Two point mutations in the α-tubulin gene at the highly conserved Arg-243 residue were identified in trifluralin-resistant *L. rigidum* plants, resulting in Arg-243-Met and Arg-243-Lys amino acid substitutions. Transgenic rice calli overexpressing these two mutant genes were resistant to trifluralin and other dinitroaniline herbicides. Structural modeling of the molecular interactions between *L*. *rigidum* α-tubulin and trifluralin predict that the trifluralin binding pocket involves amino acid residues that are in direct contact with Arg-243. Thus, mutations at the 243 position cause significant changes in the herbicide-binding domain, thereby reducing herbicide binding and conferring resistance. Together with the observation that homozygous Arg-243-Met mutants showed twisted growth, it is likely that Arg-243 is important not only for determining dinitroaniline herbicide sensitivity but also for normal microtubule function.

## Author Contributions

ZC, HH, and QY designed the experiments. ZC and JC performed the experimental work. AN performed the modeling work. SP supervised the research and manuscript preparation. All authors wrote and edited the manuscript.

## Conflict of Interest Statement

The authors declare that the research was conducted in the absence of any commercial or financial relationships that could be construed as a potential conflict of interest.

## Footnotes

1 http://ncbi.nlm.nih.gov/sra

2 http://www.accelrys.com/

## References

[B1] Abraham M. J. Murtola T. Schulz R. Páll S. Smith J. C. Hess B. (2015). GROMACS: high performance molecular simulations through multi-level parallelism from laptops to supercomputers. *SoftwareX* 1 19–25. 10.1016/j.softx.2015.06.001

[B2] Alushin G. M. Lander G. C. Kellogg E. H. Zhang R. Baker D. Nogales E. (2014). High-resolution microtubule structures reveal the structural transitions in αβ-tubulin upon GTP hydrolysis. *Cell* 157 1117–1129. 10.1016/j.cell.2014.03.053 24855948 PMC4054694

[B3] Anthony R. G. Hussey P. J. (1998). Suppression of endogenous α and β tubulin synthesis in transgenic maize calli overexpressing α and β tubulins. *Plant J.* 16 297–304. 10.1046/j.1365-313x.1998.00296.x 9881152

[B4] Anthony R. G. Hussey P. J. (1999). Double mutation in *Eleusine indica* α-tubulin increases the resistance of transgenic maize calli to dinitroaniline and phosphorothioamidate herbicides. *Plant J.* 18 669–674. 10.1046/j.1365-313x.1999.00484.x 10417718

[B5] Anthony R. G. Reichelt S. Hussey P. J. (1999). Dinitroaniline herbicide-resistant transgenic tobacco plants generated by co-overexpression of a mutant α-tubulin and a β-tubulin. *Nat. Biotechnol.* 17 712–716. 10.1038/10931 10404167

[B6] Anthony R. G. Waldin T. R. Ray J. A. Bright S. W. Hussey P. J. (1998). Herbicide resistance caused by spontaneous mutation of the cytoskeletal protein tubulin. *Nature* 393 260–263. 10.1038/30484 9607761

[B7] Bayer G. Y. Yemets A. I. Blume Y. B. (2014). Obtaining the transgenic lines of finger millet *Eleusine coracana* (L.). with dinitroaniline resistance. *Cytol. Genet.* 48 139–144. 10.3103/S0095452714030025 25016822

[B8] Blume Y. Yemets A. Sheremet Y. Nyporko A. Sulimenko V. Sulimenko T. (2010). Exposure of beta-tubulin regions defined by antibodies on an *Arabidopsis thaliana* microtubule protofilament model and in the cells. *BMC Plant Biol.* 10:29. 10.1186/1471-2229-10-29 20167106 PMC2844066

[B9] Blume Y. B. Nyporko A. Y. Yemets A. Baird W. (2003). Structural modeling of the interaction of plant α-tubulin with dinitroaniline and phosphoroamidate herbicides. *Cell Biol. Int.* 27 171–174. 10.1016/S1065-6995(02)00298-6 12681297

[B10] Blume Y. B. Strashnyuk N. M. Smertenko A. P. Solodushko V. G. Sidorov V. A. Gleba Y. Y. (1998). Alteration of β-tubulin in *Nicotiana plumbaginifolia* confers resistance to amiprophos-methyl. *Theor. Appl. Genet.* 97 464–472. 10.1007/s001220050918

[B11] Boutsalis P. Gill G. S. Preston C. (2012). Incidence of herbicide resistance in rigid ryegrass (*Lolium rigidum*) across southeastern Australia. *Weed Technol.* 26 391–398. 10.1614/WT-D-11-00150.1

[B12] Bussi G. Donadio D. Parrinello M. (2007). Canonical sampling through velocity rescaling. *J. Chem. Phys.* 126 014101. 10.1063/1.2408420 17212484

[B13] Chen J. Yu Q. Owen M. Han H. Powles S. (2017). Dinitroaniline herbicide resistance in a multiple-resistant *Lolium rigidum* population. *Pest Manag. Sci.* 10.1002/ps.4790 [Epub ahead of print]. 30848567

[B14] Darden T. York D. Pedersen L. (1993). Particle mesh Ewald: an *N* log(*N*) method for Ewald sums in large systems. *J. Chem. Phys.* 98 10089–10092. 10.1063/1.464397

[B15] Darmency H. Picard J. Wang T. (2011). Fitness costs linked to dinitroaniline resistance mutation in *Setaria*. *Heredity* 107 80–86. 10.1038/hdy.2010.169 21245896 PMC3186120

[B16] Das B. Meirovitch H. Navon I. M. (2003). Performance of hybrid methods for large-scale unconstrained optimization as applied to models of proteins. *J. Comput. Chem.* 24 1222–1231. 10.1002/jcc.10275 12820130

[B17] Délye C. Menchari Y. Michel S. Darmency H. (2004). Molecular bases for sensitivity to tubulin-binding herbicides in green foxtail. *Plant Physiol.* 136 3920–3932. 10.1104/pp.103.037432 15531712 PMC535825

[B18] Duhoux A. Carrère S. Gouzy J. Bonin L. Délye C. (2015). RNA-Seq analysis of rye-grass transcriptomic response to an herbicide inhibiting acetolactate-synthase identifies transcripts linked to non-target-site-based resistance. *Plant Mol. Biol.* 87 473–487. 10.1007/s11103-015-0292-3 25636204

[B19] Eswar N. Webb B. Marti-Renom M. A. Madhusudhan M. S. Eramian D. Shen M. Y. (2006). Comparative protein structure modeling using Modeller. *Curr. Protoc. Bioinformatics* 5 5.6.1–5.6.30. 10.1002/0471250953.bi0506s15 18428767 PMC4186674

[B20] Fleet B. Malone J. Preston C. Gill G. (2017). Target-site point mutation conferring resistance to trifluralin in rigid ryegrass (*Lolium rigidum*). *Weed Sci.* 2017 1–8. 10.1017/wsc.2017.67

[B21] Hashim S. Jan A. Sunohara Y. Hachinohe M. Ohdan H. Matsumoto H. (2012). Mutation of alpha-tubulin genes in trifluralin-resistant water foxtail (*Alopecurus aequalis*). *Pest. Manag. Sci.* 68 422–429. 10.1002/ps.2284 21972152

[B22] Heap I. (2017). *The International Survey of Herbicide Resistant Weeds.* Available at: www.weedscience.org [accessed October 14 2017].

[B23] Hockney R. W. Goel S. P. Eastwood J. W. (1974). Quiet high-resolution computer models of a plasma. *J. Comput. Phys.* 14 148–158. 10.1016/0021-9991(74)90010-2

[B24] Hoffer L. Horvath D. (2012). S4MPLE–Sampler For Multiple Protein–Ligand Entities: simultaneous docking of several entities. *J. Chem. Inf. Model.* 53 88–102. 10.1021/ci300495r 23215156

[B25] Howard J. Hyman A. A. (2003). Dynamics and mechanics of the microtubule plus end. *Nature* 422 753–758. 10.1038/nature01600 12700769

[B26] James S. W. Silflow C. D. Stroom P. Lefebvre P. A. (1993). A mutation in the alpha 1-tubulin gene of *Chlamydomonas reinhardtii* confers resistance to anti-microtubule herbicides. *J. Cell Sci.* 106 209–218. 7903670 10.1242/jcs.106.1.209

[B27] Kumari R. Kumar R. Open Source Drug Discovery Consortium Lynn A. (2014). g_mmpbsa–a GROMACS tool for high-throughput MM-PBSA calculations. *J. Chem. Inf. Model.* 54 1951–1962. 10.1021/ci500020m 24850022

[B28] Lee V. D. Huang B. (1990). Missense mutations at lysine 350 in beta 2-tubulin confer altered sensitivity to microtubule inhibitors in *Chlamydomonas*. *Plant Cell* 2 1051–1057. 10.1105/tpc.2.11.1051 2152107 PMC159953

[B29] Lyons-Abbott S. Sackett D. L. Wloga D. Gaertig J. Morgan R. E. Werbovetz K. A. (2010). α-Tubulin mutations alter oryzalin affinity and microtubule assembly properties to confer dinitroaniline resistance. *Eukaryot. Cell* 9 1825–1834. 10.1128/EC.00140-10 20870876 PMC3008275

[B30] Ma C. Li C. Ganesan L. Oak J. Tsai S. Sept D. (2007). Mutations in α-tubulin confer dinitroaniline resistance at a cost to microtubule function. *Mol. Biol. Cell* 18 4711–4720. 10.1091/mbc.E07-04-0379 17881728 PMC2096588

[B31] MacKerell A. D. Banavali N. Foloppe N. (2000). Development and current status of the CHARMM force field for nucleic acids. *Biopolymers* 56 257–265. 10.1002/1097-0282(2000)56:4<257::AID-BIP10029>3.0.CO;2-W 11754339

[B32] MacKerell A. D. Feig M. Brooks C. L. (2004). Extending the treatment of backbone energetics in protein force fields: limitations of gas-phase quantum mechanics in reproducing protein conformational distributions in molecular dynamics simulations. *J. Comput. Chem.* 25 1400–1415. 10.1002/jcc.20065 15185334

[B33] Mahoney M. W. Jorgensen W. L. (2000). A five-site model for liquid water and the reproduction of the density anomaly by rigid, nonpolarizable potential functions. *J. Chem. Phys.* 112 8910–8922. 10.1063/1.481505

[B34] McAlister F. M. Holtum J. A. Powles S. B. (1995). Dintroaniline herbicide resistance in rigid ryegrass (*Lolium rigidum*). *Weed Sci.* 43 55–62.

[B35] Morrissette N. S. Mitra A. Sept D. Sibley L. D. (2004). Dinitroanilines bind α-tubulin to disrupt microtubules. *Mol. Biol. Cell* 15 1960–1968. 10.1091/mbc.E03-07-0530 14742718 PMC379290

[B36] Nogales E. (1999). A structural view of microtubule dynamics. *Cell. Mol. Life Sci.* 56 133–142. 10.1007/s000180050012 11213253 PMC11146758

[B37] Nyporko A. Y. Blume Y. B. (2008). “Spatial distribution of tubulin mutations conferring resistance to antimicrotubular compounds,” in *The Plant Cytoskeleton: a Key Tool for Agro-Biotechnology* eds Blume Y. B. Vance Baird W. Yemets A. I. Breviario D. (Berlin: Springer) 397–417.

[B38] Nyporko A. Y. Blume Y. B. (2014). Structural mechanisms of interaction of cyanolcrylates with plant tubulin. *Cytol. Genet.* 48 7–14. 10.3103/S009545271401006X 24791469

[B39] Nyporko A. Y. Yemets A. I. Brytsun V. N. Lozinsky M. O. Blume Y. B. (2009). Structural and biological characterization of the tubulin interaction with dinitroanilines. *Cytol. Genet.* 43 267–282. 10.3103/S0095452709040082 19938648

[B40] Nyporko A. Y. Yemets A. I. Klimkina L. A. Blume Y. B. (2002). Sensitivity of *Eleusine indica* callus to trifluralin and amiprophosmethyl in correlation with the binding of these compounds to Tubulin. *Russ. J. Plant Physiol.* 49 413–418. 10.1023/a:1015561523131

[B41] Owen M. J. Martinez N. J. Powles S. B. (2014). Multiple herbicide-resistant *Lolium rigidum* (annual ryegrass) now dominates across the Western Australian grain belt. *Weed Res.* 54 314–324. 10.1111/wre.12068

[B42] Owen M. J. Walsh M. J. Llewellyn R. S. Powles S. B. (2007). Widespread occurrence of multiple herbicide resistance in Western Australian annual ryegrass (*Lolium rigidum*) populations. *Crop Pasture Sci.* 58 711–718. 10.1071/AR06283 16906433

[B43] Sunohara H. Kawai T. Shimizu-Sato S. Sato Y. Sato K. Kitano H. (2009). A dominant mutation of TWISTED DWARF 1 encoding an α-tubulin protein causes severe dwarfism and right helical growth in rice. *Genes Genet. Syst.* 84 209–218. 10.1266/ggs.84.209 19745569

[B44] Venselaar H. Joosten R. P. Vroling B. Baakman C. A. B. Hekkelman M. L. Krieger E. (2010). Homology modelling and spectroscopy, a never-ending love story. *Eur. Biophys. J.* 39 551–563. 10.1007/s00249-009-0531-0 19718498 PMC2841279

[B45] Yamamoto E. Zeng L. Baird W. V. (1998). α-Tubulin missense mutations correlate with antimicrotubule drug resistance in *Eleusine indica*. *Plant Cell* 10 297–308. 10.1105/tpc.10.2.297 9490751 PMC143984

[B46] Zhao J. Chen H. Ren D. Tang H. Qiu R. Feng J. (2015). Genetic interactions between diverged alleles of Early heading date 1 (Ehd1) and Heading date 3a (Hd3a)/RICE FLOWERING LOCUS T1 (RFT1) control differential heading and contribute to regional adaptation in rice (*Oryza sativa*). *New Phytol.* 208 936–948. 10.1111/nph.13503 26096631

[B47] Zoete V. Cuendet M. A. Grosdidier A. Michielin O. (2011). SwissParam: a fast force field generation tool for small organic molecules. *J. Comput. Chem.* 32 2359–2368. 10.1002/jcc.21816 21541964

